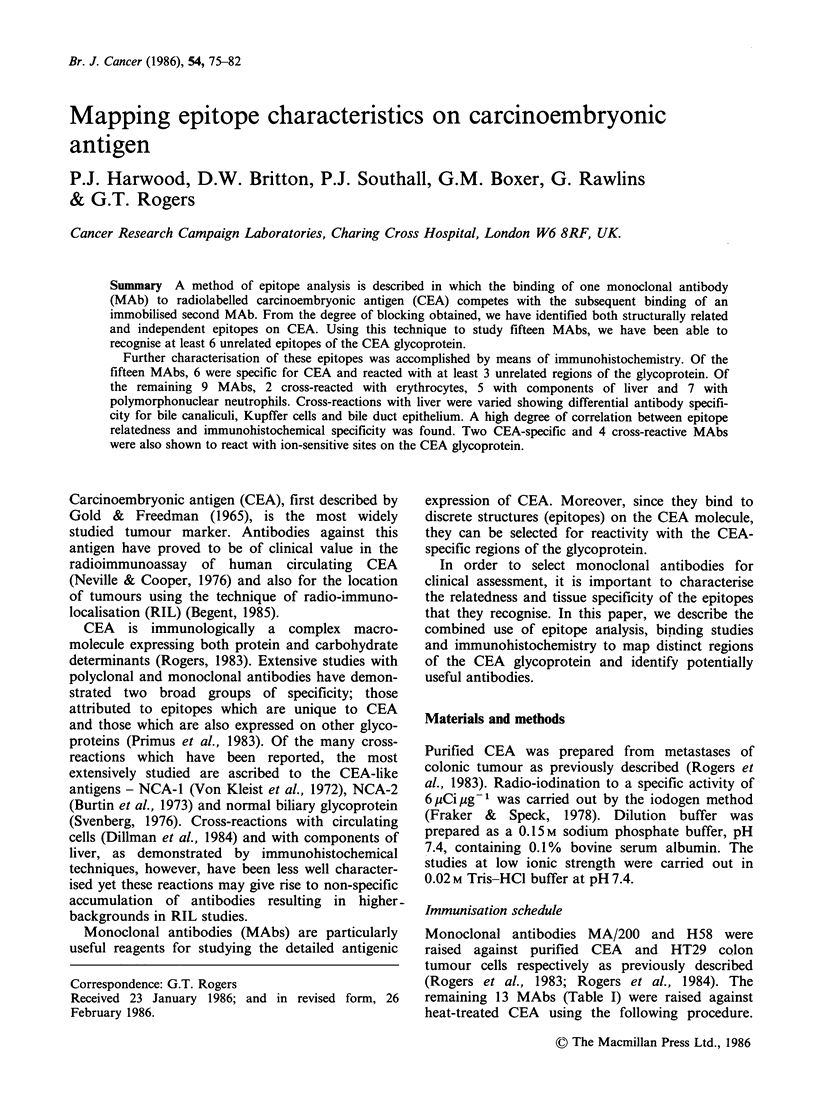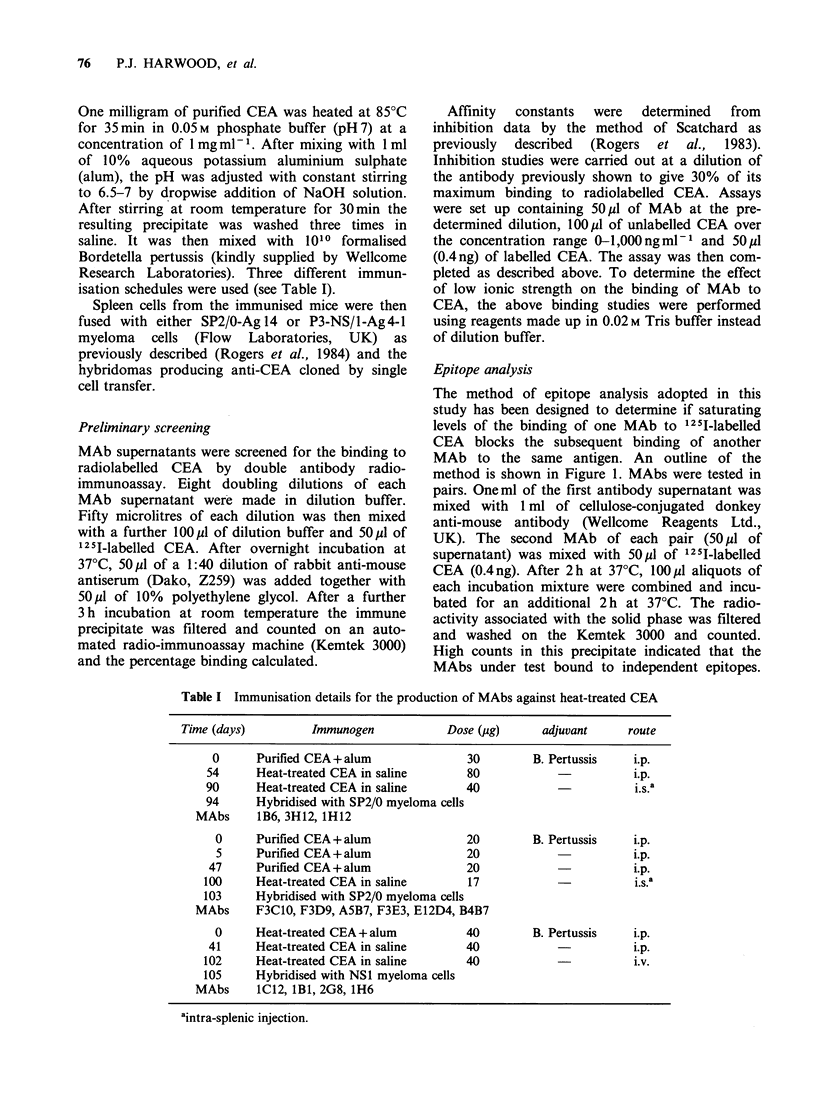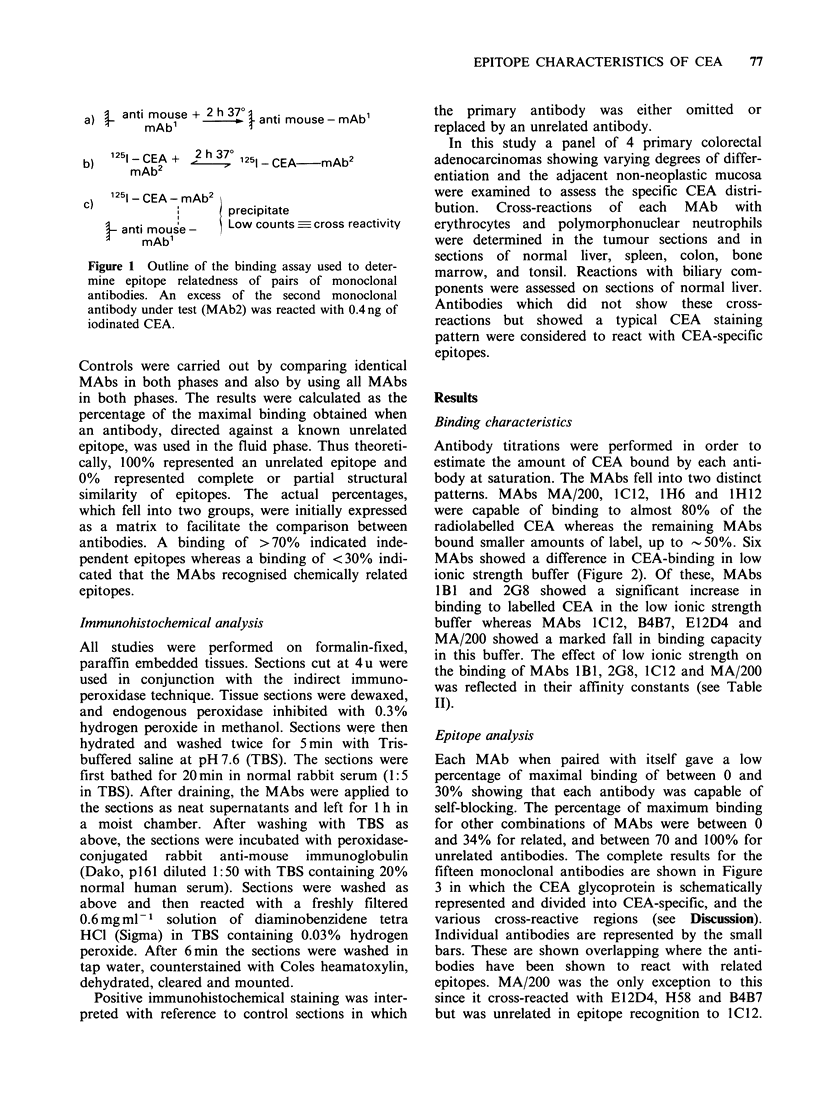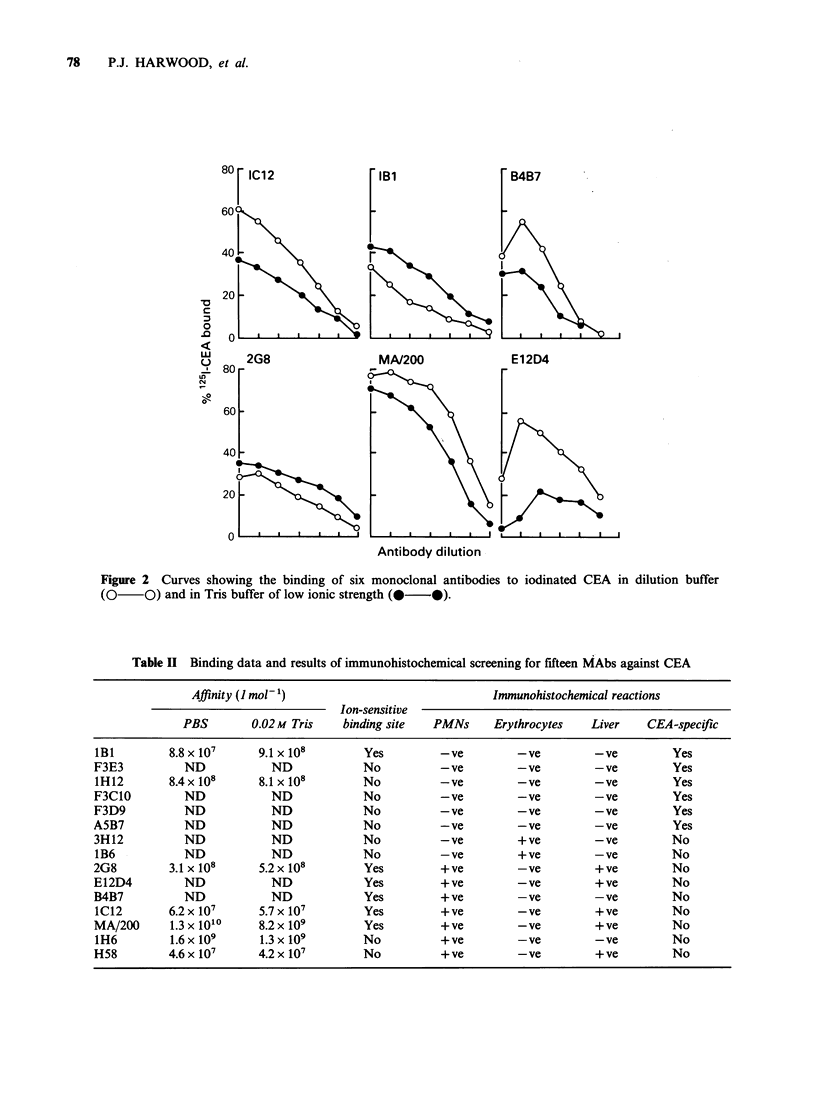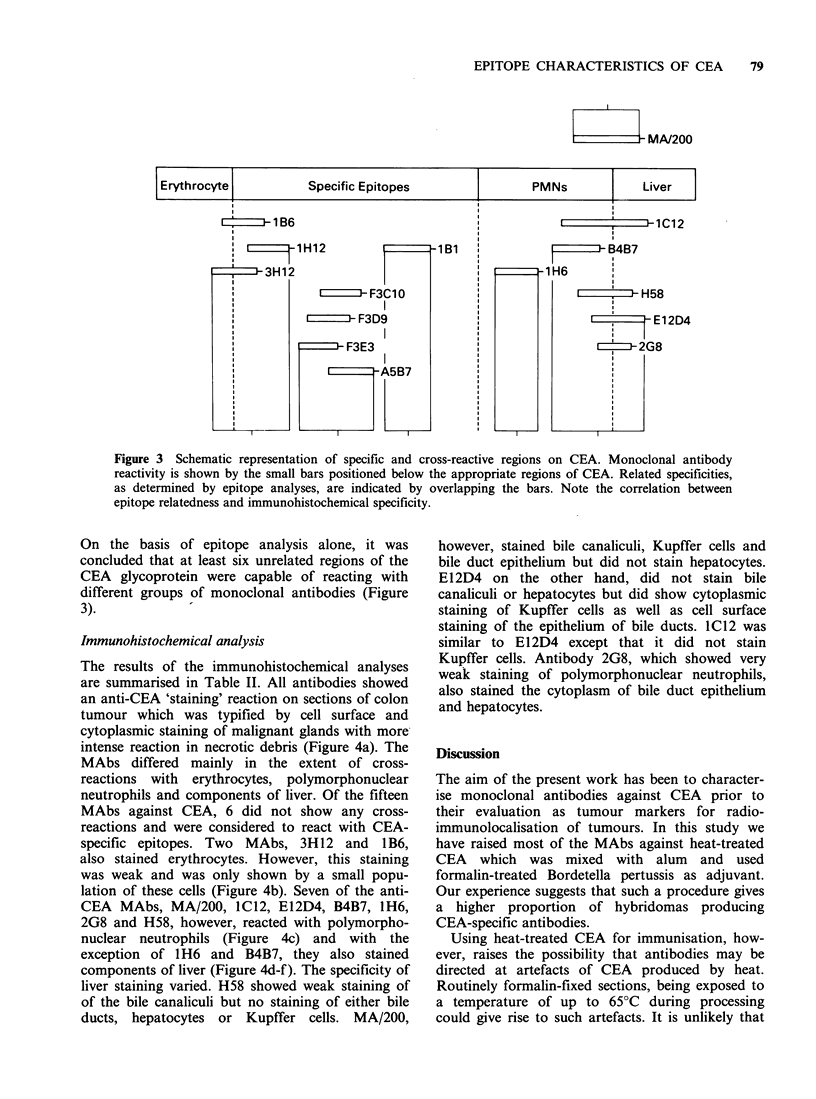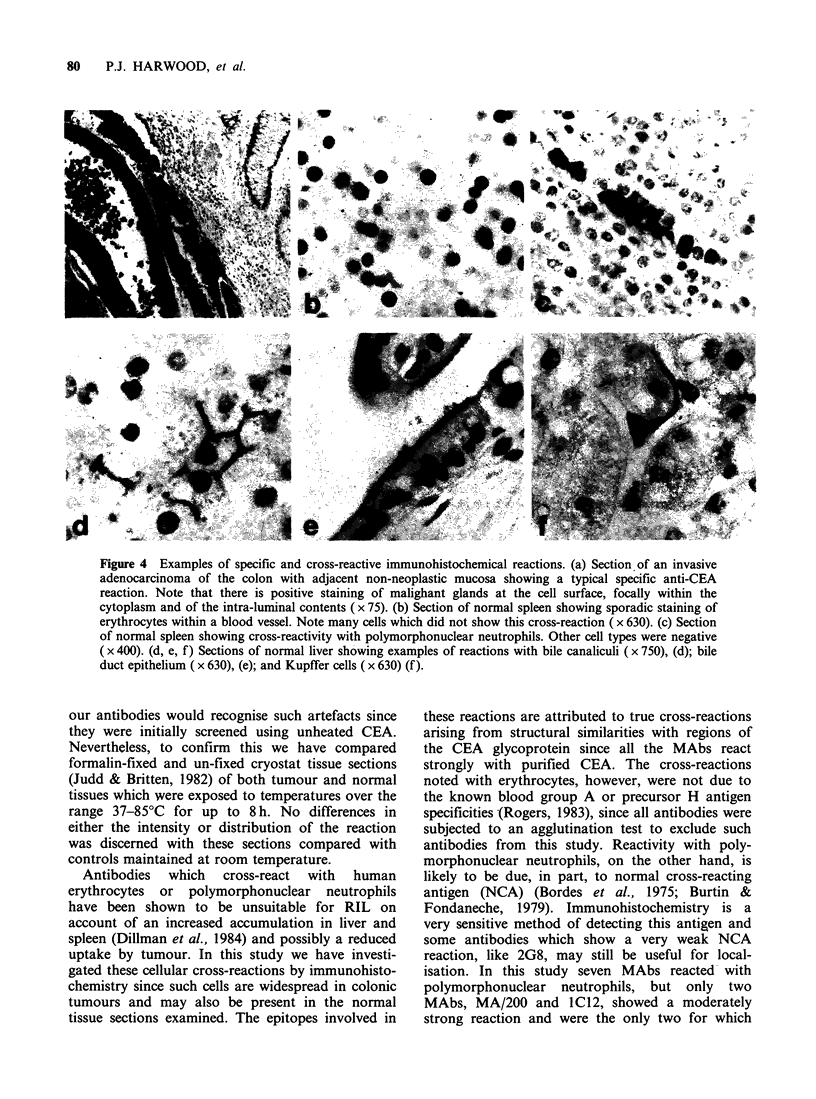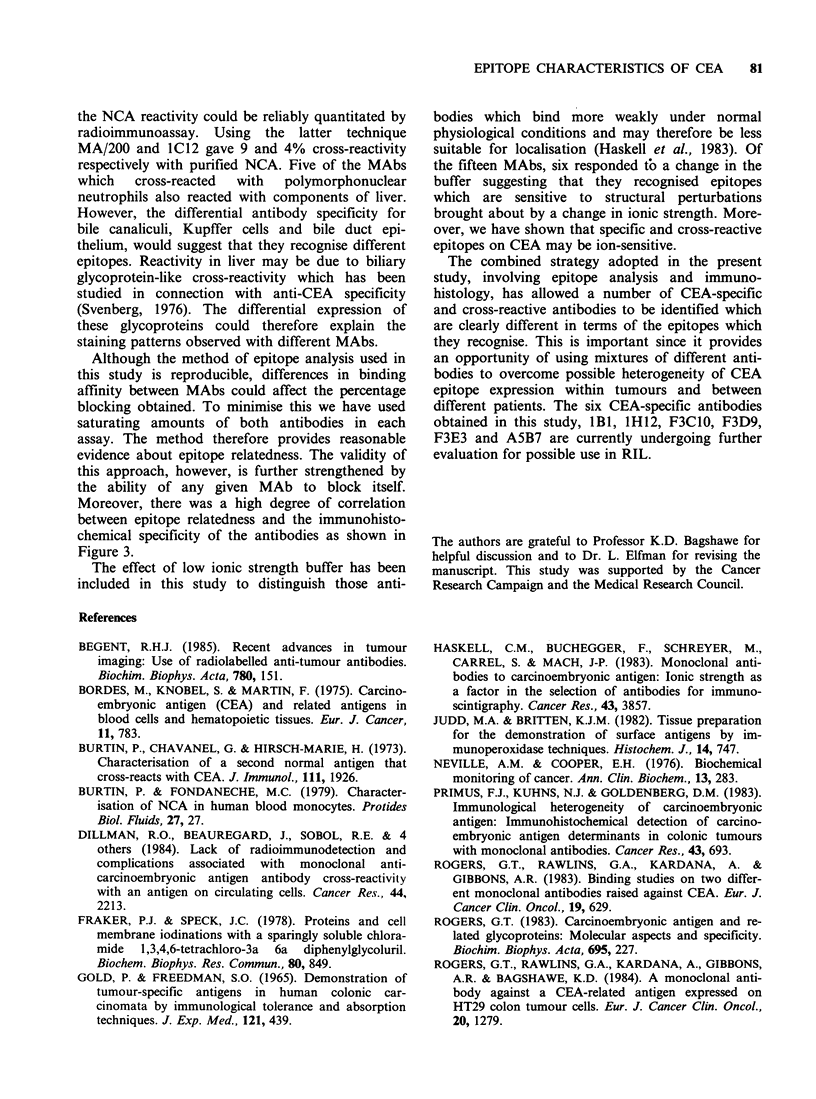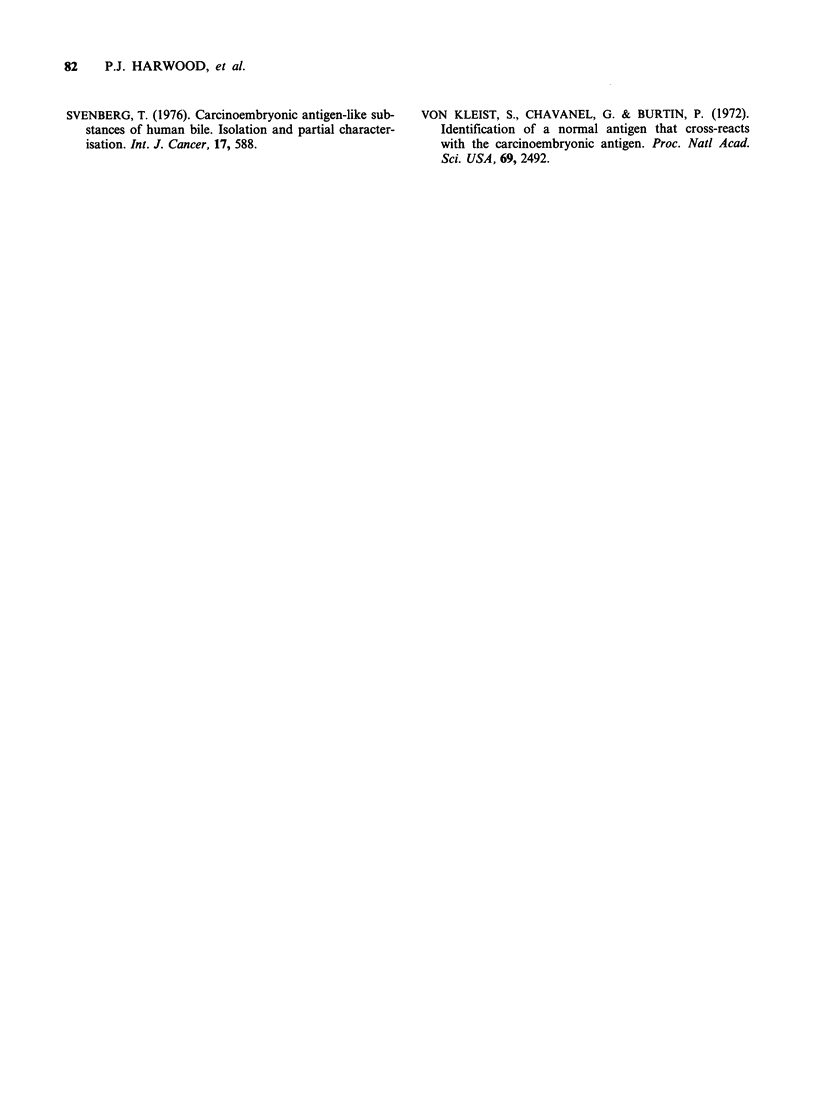# Mapping epitope characteristics on carcinoembryonic antigen.

**DOI:** 10.1038/bjc.1986.154

**Published:** 1986-07

**Authors:** P. J. Harwood, D. W. Britton, P. J. Southall, G. M. Boxer, G. Rawlins, G. T. Rogers

## Abstract

**Images:**


*Br. J. Cancer* (1986), **54**, 75–82

### Mapping epitope characteristics on carcinoembryonic antigen

P.J. Harwood, D.W. Britton, P.J. Southall, G.M. Boxer, G. Rawlins & G.T. Rogers

*Cancer Research Campaign Laboratories, Charing Cross Hospital, London W6 8RF, UK.*

**Summary** A method of epitope analysis is described in which the binding of one monoclonal antibody (MAb) to radiolabelled carcinoembryonic antigen (CEA) competes with the subsequent binding of an immobilised second MAb. From the degree of blocking obtained, we have identified both structurally related and independent epitopes on CEA. Using this technique to study fifteen MAbs, we have been able to recognise at least 6 unrelated epitopes of the CEA glycoprotein.

Further characterisation of these epitopes was accomplished by means of immunohistochemistry. Of the fifteen MAbs, 6 were specific for CEA and reacted with at least 3 unrelated regions of the glycoprotein. Of the remaining 9 MAbs, 2 cross-reacted with erythrocytes, 5 with components of liver and 7 with polymorphonuclear neutrophils. Cross-reactions with liver were varied showing differential antibody specificity for bile canaliculi, Kupffer cells and bile duct epithelium. A high degree of correlation between epitope relatedness and immunohistochemical specificity was found. Two CEA-specific and 4 cross-reactive MAbs were also shown to react with ion-sensitive sites on the CEA glycoprotein.

Carcinoembryonic antigen (CEA), first described by Gold & Freedman (1965), is the most widely studied tumour marker. Antibodies against this antigen have proved to be of clinical value in the radioimmunoassay of human circulating CEA (Neville & Cooper, 1976) and also for the location of tumours using the technique of radio-immuno-localisation (RIL) (Begent, 1985).

CEA is immunologically a complex macro-molecule expressing both protein and carbohydrate determinants (Rogers, 1983). Extensive studies with polyclonal and monoclonal antibodies have demonstrated two broad groups of specificity; those attributed to epitopes which are unique to CEA and those which are also expressed on other glyco-proteins (Primus *et al.*, 1983). Of the many cross-reactions which have been reported, the most extensively studied are ascribed to the CEA-like antigens – NCA-1 (Von Kleist *et al.*, 1972), NCA-2 (Burtin *et al.*, 1973) and normal biliary glycoprotein (Svenberg, 1976). Cross-reactions with circulating cells (Dillman *et al.*, 1984) and with components of liver, as demonstrated by immunohistochemical techniques, however, have been less well characterised yet these reactions may give rise to non-specific accumulation of antibodies resulting in higher backgrounds in RIL studies.

Monoclonal antibodies (MAbs) are particularly useful reagents for studying the detailed antigenic expression of CEA. Moreover, since they bind to discrete structures (epitopes) on the CEA molecule, they can be selected for reactivity with the CEA-specific regions of the glycoprotein.

In order to select monoclonal antibodies for clinical assessment, it is important to characterise the relatedness and tissue specificity of the epitopes that they recognise. In this paper, we describe the combined use of epitope analysis, binding studies and immunohistochemistry to map distinct regions of the CEA glycoprotein and identify potentially useful antibodies.

### Materials and methods

Purified CEA was prepared from metastases of colonic tumour as previously described (Rogers *et al.*, 1983). Radio-iodination to a specific activity of $6\,\mu Ci\,\mu g^{-1}$ was carried out by the iodogen method (Fraker & Speck, 1978). Dilution buffer was prepared as a 0.15 M sodium phosphate buffer, pH 7.4, containing 0.1% bovine serum albumin. The studies at low ionic strength were carried out in 0.02 M Tris–HCl buffer at pH 7.4.

### *Immunisation schedule*

Monoclonal antibodies MA/200 and H58 were raised against purified CEA and HT29 colon tumour cells respectively as previously described (Rogers *et al.*, 1983; Rogers *et al.*, 1984). The remaining 13 MAbs (Table I) were raised against heat-treated CEA using the following procedure.

Correspondence: G.T. Rogers
Received 23 January 1986; and in revised form, 26 February 1986.

© The Macmillan Press Ltd., 1986

One milligram of purified CEA was heated at 85°C for 35 min in 0.05 M phosphate buffer (pH 7) at a concentration of $1\,mg\,ml^{-1}$. After mixing with 1 ml of 10% aqueous potassium aluminium sulphate (alum), the pH was adjusted with constant stirring to 6.5–7 by dropwise addition of NaOH solution. After stirring at room temperature for 30 min the resulting precipitate was washed three times in saline. It was then mixed with $10^{10}$ formalised Bordetella pertussis (kindly supplied by Wellcome Research Laboratories). Three different immunisation schedules were used (see Table I).

Spleen cells from the immunised mice were then fused with either SP2/0-Ag 14 or P3-NS/1-Ag 4-1 myeloma cells (Flow Laboratories, UK) as previously described (Rogers *et al.*, 1984) and the hybridomas producing anti-CEA cloned by single cell transfer.

*Preliminary screening*

MAb supernatants were screened for the binding to radiolabelled CEA by double antibody radioimmunoassay. Eight doubling dilutions of each MAb supernatant were made in dilution buffer. Fifty microlitres of each dilution was then mixed with a further 100 µl of dilution buffer and 50 µl of $^{125}$I-labelled CEA. After overnight incubation at 37°C, 50 µl of a 1:40 dilution of rabbit anti-mouse antiserum (Dako, Z259) was added together with 50 µl of 10% polyethylene glycol. After a further 3 h incubation at room temperature the immune precipitate was filtered and counted on an automated radio-immunoassay machine (Kemtek 3000) and the percentage binding calculated.

Affinity constants were determined from inhibition data by the method of Scatchard as previously described (Rogers *et al.*, 1983). Inhibition studies were carried out at a dilution of the antibody previously shown to give 30% of its maximum binding to radiolabelled CEA. Assays were set up containing 50 µl of MAb at the predetermined dilution, 100 µl of unlabelled CEA over the concentration range 0–1,000 $ng\,ml^{-1}$ and 50 µl (0.4 ng) of labelled CEA. The assay was then completed as described above. To determine the effect of low ionic strength on the binding of MAb to CEA, the above binding studies were performed using reagents made up in 0.02 M Tris buffer instead of dilution buffer.

*Epitope analysis*

The method of epitope analysis adopted in this study has been designed to determine if saturating levels of the binding of one MAb to $^{125}$I-labelled CEA blocks the subsequent binding of another MAb to the same antigen. An outline of the method is shown in Figure 1. MAbs were tested in pairs. One ml of the first antibody supernatant was mixed with 1 ml of cellulose-conjugated donkey anti-mouse antibody (Wellcome Reagents Ltd., UK). The second MAb of each pair (50 µl of supernatant) was mixed with 50 µl of $^{125}$I-labelled CEA (0.4 ng). After 2 h at 37°C, 100 µl aliquots of each incubation mixture were combined and incubated for an additional 2 h at 37°C. The radioactivity associated with the solid phase was filtered and washed on the Kemtek 3000 and counted. High counts in this precipitate indicated that the MAbs under test bound to independent epitopes.

**Table I** Immunisation details for the production of MAbs against heat-treated CEA

| *Time (days)* | *Immunogen* | *Dose (µg)* | *adjuvant* | *route* |
|---|---|---|---|---|
| 0 | Purified CEA + alum | 30 | B. Pertussis | i.p. |
| 54 | Heat-treated CEA in saline | 80 | — | i.p. |
| 90 | Heat-treated CEA in saline | 40 | — | i.s.[a] |
| 94 | Hybridised with SP2/0 myeloma cells | | | |
| MAbs | 1B6, 3H12, 1H12 | | | |
| 0 | Purified CEA + alum | 20 | B. Pertussis | i.p. |
| 5 | Purified CEA + alum | 20 | — | i.p. |
| 47 | Purified CEA + alum | 20 | — | i.p. |
| 100 | Heat-treated CEA in saline | 17 | — | i.s.[a] |
| 103 | Hybridised with SP2/0 myeloma cells | | | |
| MAbs | F3C10, F3D9, A5B7, F3E3, E12D4, B4B7 | | | |
| 0 | Heat-treated CEA + alum | 40 | B. Pertussis | i.p. |
| 41 | Heat-treated CEA in saline | 40 | — | i.p. |
| 102 | Heat-treated CEA in saline | 40 | — | i.v. |
| 105 | Hybridised with NS1 myeloma cells | | | |
| MAbs | 1C12, 1B1, 2G8, 1H6 | | | |

[a]intra-splenic injection.

**Figure 1** Outline of the binding assay used to determine epitope relatedness of pairs of monoclonal antibodies. An excess of the second monoclonal antibody under test (MAb2) was reacted with 0.4 ng of iodinated CEA.

Controls were carried out by comparing identical MAbs in both phases and also by using all MAbs in both phases. The results were calculated as the percentage of the maximal binding obtained when an antibody, directed against a known unrelated epitope, was used in the fluid phase. Thus theoretically, 100% represented an unrelated epitope and 0% represented complete or partial structural similarity of epitopes. The actual percentages, which fell into two groups, were initially expressed as a matrix to facilitate the comparison between antibodies. A binding of >70% indicated independent epitopes whereas a binding of <30% indicated that the MAbs recognised chemically related epitopes.

*Immunohistochemical analysis*

All studies were performed on formalin-fixed, paraffin embedded tissues. Sections cut at 4 u were used in conjunction with the indirect immunoperoxidase technique. Tissue sections were dewaxed, and endogenous peroxidase inhibited with 0.3% hydrogen peroxide in methanol. Sections were then hydrated and washed twice for 5 min with Tris-buffered saline at pH 7.6 (TBS). The sections were first bathed for 20 min in normal rabbit serum (1:5 in TBS). After draining, the MAbs were applied to the sections as neat supernatants and left for 1 h in a moist chamber. After washing with TBS as above, the sections were incubated with peroxidase-conjugated rabbit anti-mouse immunoglobulin (Dako, p161 diluted 1:50 with TBS containing 20% normal human serum). Sections were washed as above and then reacted with a freshly filtered 0.6 mg ml$^{-1}$ solution of diaminobenzidene tetra HCl (Sigma) in TBS containing 0.03% hydrogen peroxide. After 6 min the sections were washed in tap water, counterstained with Coles heamatoxylin, dehydrated, cleared and mounted.

Positive immunohistochemical staining was interpreted with reference to control sections in which the primary antibody was either omitted or replaced by an unrelated antibody.

In this study a panel of 4 primary colorectal adenocarcinomas showing varying degrees of differentiation and the adjacent non-neoplastic mucosa were examined to assess the specific CEA distribution. Cross-reactions of each MAb with erythrocytes and polymorphonuclear neutrophils were determined in the tumour sections and in sections of normal liver, spleen, colon, bone marrow, and tonsil. Reactions with biliary components were assessed on sections of normal liver. Antibodies which did not show these cross-reactions but showed a typical CEA staining pattern were considered to react with CEA-specific epitopes.

### Results

*Binding characteristics*

Antibody titrations were performed in order to estimate the amount of CEA bound by each antibody at saturation. The MAbs fell into two distinct patterns. MAbs MA/200, 1C12, 1H6 and 1H12 were capable of binding to almost 80% of the radiolabelled CEA whereas the remaining MAbs bound smaller amounts of label, up to ~50%. Six MAbs showed a difference in CEA-binding in low ionic strength buffer (Figure 2). Of these, MAbs 1B1 and 2G8 showed a significant increase in binding to labelled CEA in the low ionic strength buffer whereas MAbs 1C12, B4B7, E12D4 and MA/200 showed a marked fall in binding capacity in this buffer. The effect of low ionic strength on the binding of MAbs 1B1, 2G8, 1C12 and MA/200 was reflected in their affinity constants (see Table II).

*Epitope analysis*

Each MAb when paired with itself gave a low percentage of maximal binding of between 0 and 30% showing that each antibody was capable of self-blocking. The percentage of maximum binding for other combinations of MAbs were between 0 and 34% for related, and between 70 and 100% for unrelated antibodies. The complete results for the fifteen monoclonal antibodies are shown in Figure 3 in which the CEA glycoprotein is schematically represented and divided into CEA-specific, and the various cross-reactive regions (see **Discussion**). Individual antibodies are represented by the small bars. These are shown overlapping where the antibodies have been shown to react with related epitopes. MA/200 was the only exception to this since it cross-reacted with E12D4, H58 and B4B7 but was unrelated in epitope recognition to 1C12.

**Figure 2** Curves showing the binding of six monoclonal antibodies to iodinated CEA in dilution buffer (○——○) and in Tris buffer of low ionic strength (●——●).

**Table II** Binding data and results of immunohistochemical screening for fifteen MAbs against CEA

| | *Affinity ($l\ mol^{-1}$)* | | *Ion-sensitive binding site* | *Immunohistochemical reactions* | | | |
|---|---|---|---|---|---|---|---|
| | *PBS* | *0.02* M *Tris* | | *PMNs* | *Erythrocytes* | *Liver* | *CEA-specific* |
| 1B1 | $8.8 \times 10^7$ | $9.1 \times 10^8$ | Yes | −ve | −ve | −ve | Yes |
| F3E3 | ND | ND | No | −ve | −ve | −ve | Yes |
| 1H12 | $8.4 \times 10^8$ | $8.1 \times 10^8$ | No | −ve | −ve | −ve | Yes |
| F3C10 | ND | ND | No | −ve | −ve | −ve | Yes |
| F3D9 | ND | ND | No | −ve | −ve | −ve | Yes |
| A5B7 | ND | ND | No | −ve | −ve | −ve | Yes |
| 3H12 | ND | ND | No | −ve | +ve | −ve | No |
| 1B6 | ND | ND | No | −ve | +ve | −ve | No |
| 2G8 | $3.1 \times 10^8$ | $5.2 \times 10^8$ | Yes | +ve | −ve | +ve | No |
| E12D4 | ND | ND | Yes | +ve | −ve | +ve | No |
| B4B7 | ND | ND | Yes | +ve | −ve | −ve | No |
| 1C12 | $6.2 \times 10^7$ | $5.7 \times 10^7$ | Yes | +ve | −ve | +ve | No |
| MA/200 | $1.3 \times 10^{10}$ | $8.2 \times 10^9$ | Yes | +ve | −ve | +ve | No |
| 1H6 | $1.6 \times 10^9$ | $1.3 \times 10^9$ | No | +ve | −ve | −ve | No |
| H58 | $4.6 \times 10^7$ | $4.2 \times 10^7$ | No | +ve | −ve | +ve | No |

**Figure 3** Schematic representation of specific and cross-reactive regions on CEA. Monoclonal antibody reactivity is shown by the small bars positioned below the appropriate regions of CEA. Related specificities, as determined by epitope analyses, are indicated by overlapping the bars. Note the correlation between epitope relatedness and immunohistochemical specificity.

On the basis of epitope analysis alone, it was concluded that at least six unrelated regions of the CEA glycoprotein were capable of reacting with different groups of monoclonal antibodies (Figure 3).

### *Immunohistochemical analysis*

The results of the immunohistochemical analyses are summarised in Table II. All antibodies showed an anti-CEA 'staining' reaction on sections of colon tumour which was typified by cell surface and cytoplasmic staining of malignant glands with more intense reaction in necrotic debris (Figure 4a). The MAbs differed mainly in the extent of cross-reactions with erythrocytes, polymorphonuclear neutrophils and components of liver. Of the fifteen MAbs against CEA, 6 did not show any cross-reactions and were considered to react with CEA-specific epitopes. Two MAbs, 3H12 and 1B6, also stained erythrocytes. However, this staining was weak and was only shown by a small population of these cells (Figure 4b). Seven of the anti-CEA MAbs, MA/200, 1C12, E12D4, B4B7, 1H6, 2G8 and H58, however, reacted with polymorphonuclear neutrophils (Figure 4c) and with the exception of 1H6 and B4B7, they also stained components of liver (Figure 4d-f). The specificity of liver staining varied. H58 showed weak staining of of the bile canaliculi but no staining of either bile ducts, hepatocytes or Kupffer cells. MA/200, however, stained bile canaliculi, Kupffer cells and bile duct epithelium but did not stain hepatocytes. E12D4 on the other hand, did not stain bile canaliculi or hepatocytes but did show cytoplasmic staining of Kupffer cells as well as cell surface staining of the epithelium of bile ducts. 1C12 was similar to E12D4 except that it did not stain Kupffer cells. Antibody 2G8, which showed very weak staining of polymorphonuclear neutrophils, also stained the cytoplasm of bile duct epithelium and hepatocytes.

### Discussion

The aim of the present work has been to characterise monoclonal antibodies against CEA prior to their evaluation as tumour markers for radioimmunolocalisation of tumours. In this study we have raised most of the MAbs against heat-treated CEA which was mixed with alum and used formalin-treated Bordetella pertussis as adjuvant. Our experience suggests that such a procedure gives a higher proportion of hybridomas producing CEA-specific antibodies.

Using heat-treated CEA for immunisation, however, raises the possibility that antibodies may be directed at artefacts of CEA produced by heat. Routinely formalin-fixed sections, being exposed to a temperature of up to 65°C during processing could give rise to such artefacts. It is unlikely that

**Figure 4** Examples of specific and cross-reactive immunohistochemical reactions. (a) Section of an invasive adenocarcinoma of the colon with adjacent non-neoplastic mucosa showing a typical specific anti-CEA reaction. Note that there is positive staining of malighant glands at the cell surface, focally within the cytoplasm and of the intra-luminal contents ( x 75). (b) Section of normal spleen showing sporadic staining of erythrocytes within a blood vessel. Note many cells which did not show this cross-reaction ( x 630). (c) Section of normal spleen showing cross-reactivity with polymorphonuclear neutrophils. Other cell types were negative ( x 400). (d, e, f) Sections of normal liver showing examples of reactions with bile canaliculi ( x 750), (d); bile duct epithelium ( x 630), (e); and Kupffer cells ( x 630) (f).

our antibodies would recognise such artefacts since they were initially screened using unheated CEA. Nevertheless, to confirm this we have compared formalin-fixed and un-fixed cryostat tissue sections (Judd & Britten, 1982) of both tumour and normal tissues which were exposed to temperatures over the range 37–85°C for up to 8 h. No differences in either the intensity or distribution of the reaction was discerned with these sections compared with controls maintained at room temperature.

Antibodies which cross-react with human erythrocytes or polymorphonuclear neutrophils have been shown to be unsuitable for RIL on account of an increased accumulation in liver and spleen (Dillman *et al.*, 1984) and possibly a reduced uptake by tumour. In this study we have investigated these cellular cross-reactions by immunohistochemistry since such cells are widespread in colonic tumours and may also be present in the normal tissue sections examined. The epitopes involved in these reactions are attributed to true cross-reactions arising from structural similarities with regions of the CEA glycoprotein since all the MAbs react strongly with purified CEA. The cross-reactions noted with erythrocytes, however, were not due to the known blood group A or precursor H antigen specificities (Rogers, 1983), since all antibodies were subjected to an agglutination test to exclude such antibodies from this study. Reactivity with polymorphonuclear neutrophils, on the other hand, is likely to be due, in part, to normal cross-reacting antigen (NCA) (Bordes *et al.*, 1975; Burtin & Fondaneche, 1979). Immunohistochemistry is a very sensitive method of detecting this antigen and some antibodies which show a very weak NCA reaction, like 2G8, may still be useful for localisation. In this study seven MAbs reacted with polymorphonuclear neutrophils, but only two MAbs, MA/200 and 1C12, showed a moderately strong reaction and were the only two for which the NCA reactivity could be reliably quantitated by radioimmunoassay. Using the latter technique MA/200 and 1C12 gave 9 and 4% cross-reactivity respectively with purified NCA. Five of the MAbs which cross-reacted with polymorphonuclear neutrophils also reacted with components of liver. However, the differential antibody specificity for bile canaliculi, Kupffer cells and bile duct epithelium, would suggest that they recognise different epitopes. Reactivity in liver may be due to biliary glycoprotein-like cross-reactivity which has been studied in connection with anti-CEA specificity (Svenberg, 1976). The differential expression of these glycoproteins could therefore explain the staining patterns observed with different MAbs.

Although the method of epitope analysis used in this study is reproducible, differences in binding affinity between MAbs could affect the percentage blocking obtained. To minimise this we have used saturating amounts of both antibodies in each assay. The method therefore provides reasonable evidence about epitope relatedness. The validity of this approach, however, is further strengthened by the ability of any given MAb to block itself. Moreover, there was a high degree of correlation between epitope relatedness and the immunohistochemical specificity of the antibodies as shown in Figure 3.

The effect of low ionic strength buffer has been included in this study to distinguish those antibodies which bind more weakly under normal physiological conditions and may therefore be less suitable for localisation (Haskell *et al.*, 1983). Of the fifteen MAbs, six responded to a change in the buffer suggesting that they recognised epitopes which are sensitive to structural perturbations brought about by a change in ionic strength. Moreover, we have shown that specific and cross-reactive epitopes on CEA may be ion-sensitive.

The combined strategy adopted in the present study, involving epitope analysis and immunohistology, has allowed a number of CEA-specific and cross-reactive antibodies to be identified which are clearly different in terms of the epitopes which they recognise. This is important since it provides an opportunity of using mixtures of different antibodies to overcome possible heterogeneity of CEA epitope expression within tumours and between different patients. The six CEA-specific antibodies obtained in this study, 1B1, 1H12, F3C10, F3D9, F3E3 and A5B7 are currently undergoing further evaluation for possible use in RIL.

The authors are grateful to Professor K.D. Bagshawe for helpful discussion and to Dr. L. Elfman for revising the manuscript. This study was supported by the Cancer Research Campaign and the Medical Research Council.

**References**

BEGENT, R.H.J. (1985). Recent advances in tumour imaging: Use of radiolabelled anti-tumour antibodies. *Biochim. Biophys. Acta*, **780**, 151.

BORDES, M., KNOBEL, S. & MARTIN, F. (1975). Carcinoembryonic antigen (CEA) and related antigens in blood cells and hematopoietic tissues. *Eur. J. Cancer*, **11**, 783.

BURTIN, P., CHAVANEL, G. & HIRSCH-MARIE, H. (1973). Characterisation of a second normal antigen that cross-reacts with CEA. *J. Immunol.*, **111**, 1926.

BURTIN, P. & FONDANECHE, M.C. (1979). Characterisation of NCA in human blood monocytes. *Protides Biol. Fluids*, **27**, 27.

DILLMAN, R.O., BEAUREGARD, J., SOBOL, R.E. & 4 others (1984). Lack of radioimmunodetection and complications associated with monoclonal anti-carcinoembryonic antigen antibody cross-reactivity with an antigen on circulating cells. *Cancer Res.*, **44**, 2213.

FRAKER, P.J. & SPECK, J.C. (1978). Proteins and cell membrane iodinations with a sparingly soluble chloramide 1,3,4,6-tetrachloro-3a 6a diphenylglycoluril. *Biochem. Biophys. Res. Commun.*, **80**, 849.

GOLD, P. & FREEDMAN, S.O. (1965). Demonstration of tumour-specific antigens in human colonic carcinomata by immunological tolerance and absorption techniques. *J. Exp. Med.*, **121**, 439.

HASKELL, C.M., BUCHEGGER, F., SCHREYER, M., CARREL, S. & MACH, J-P. (1983). Monoclonal antibodies to carcinoembryonic antigen: Ionic strength as a factor in the selection of antibodies for immunoscintigraphy. *Cancer Res.*, **43**, 3857.

JUDD, M.A. & BRITTEN, K.J.M. (1982). Tissue preparation for the demonstration of surface antigens by immunoperoxidase techniques. *Histochem. J.*, **14**, 747.

NEVILLE, A.M. & COOPER, E.H. (1976). Biochemical monitoring of cancer. *Ann. Clin. Biochem.*, **13**, 283.

PRIMUS, F.J., KUHNS, N.J. & GOLDENBERG, D.M. (1983). Immunological heterogeneity of carcinoembryonic antigen: Immunohistochemical detection of carcinoembryonic antigen determinants in colonic tumours with monoclonal antibodies. *Cancer Res.*, **43**, 693.

ROGERS, G.T., RAWLINS, G.A., KARDANA, A. & GIBBONS, A.R. (1983). Binding studies on two different monoclonal antibodies raised against CEA. *Eur. J. Cancer Clin. Oncol.*, **19**, 629.

ROGERS, G.T. (1983). Carcinoembryonic antigen and related glycoproteins: Molecular aspects and specificity. *Biochim. Biophys. Acta*, **695**, 227.

ROGERS, G.T., RAWLINS, G.A., KARDANA, A., GIBBONS, A.R. & BAGSHAWE, K.D. (1984). A monoclonal antibody against a CEA-related antigen expressed on HT29 colon tumour cells. *Eur. J. Cancer Clin. Oncol.*, **20**, 1279.

SVENBERG, T. (1976). Carcinoembryonic antigen-like substances of human bile. Isolation and partial characterisation. *Int. J. Cancer,* **17,** 588.

VON KLEIST, S., CHAVANEL, G. & BURTIN, P. (1972). Identification of a normal antigen that cross-reacts with the carcinoembryonic antigen. *Proc. Natl Acad. Sci. USA,* **69,** 2492.